# Hope and Exploitation in Commercial Provision of Assisted Reproductive Technologies

**DOI:** 10.1002/hast.1513

**Published:** 2023-11-14

**Authors:** Anthony Wrigley, Gabriel Watts, Wendy Lipworth, Ainsley J. Newson

**Keywords:** hope, exploitation, assisted reproductive technologies, physician‐patient relationship, bioethics

## Abstract

*Innovation is a key driver of care provision in assisted reproductive technologies (ART). ART providers offer a range of add‐on interventions, aiming to augment standard in vitro fertilization protocols and improve the chances of a live birth. Particularly in the context of commercial provision, an ever‐increasing array of add‐ons are marketed to ART patients, even when evidence to support them is equivocal. A defining feature of ART is hope—hope that a cycle will lead to a baby or that another test or intervention will make a difference. Yet such hope also leaves ART patients vulnerable in a variety of ways. This article argues that previous attempts to safeguard ART patients have neglected how the use of add‐ons in commercial ART can exploit patients’ hopes. Commercial providers of ART should provide add‐ons only free of charge, under a suitable research protocol*.

## Article

Innovation is a key driver of care provision in medicine and health care, and assisted reproductive technologies (ART) are a case in point. Since the birth of Louise Brown—the first baby to be born as a result of in vitro fertilization (IVF)—in 1978, assisted reproduction has grown to encompass a vast range of techniques, tests, and interventions. ART is now a global enterprise, with rapidly growing markets in low‐ and middle‐income countries and well‐established provision in high‐income settings. ART has a significant commercial presence, with clinics listed on stock exchanges and venture capital and private equity investments in the sector.^1^ In this commercialized environment, undergoing ART generates significant out‐of‐pocket costs for patients^2^ in most countries.

Despite over forty years of ART, success rates remain modest. The probability that a treatment cycle will lead to a live birth varies with the age of the person receiving treatment, but remains at around 24 percent per cycle on average.^3^ Efforts to both combat this issue and gain a competitive commercial edge have driven the development of additional interventions—known as “adjuncts” or “add‐ons”—that are offered with the aim of improving the chances of a live birth.^4^ There are now many add‐ons offered in ART, including tests for patients (such as immunological screening), surgical and nonsurgical procedures (such as endometrial scratching or immune‐modulating therapies), imaging (such as timelapse imaging), in vitro interventions (such as preimplantation genetic testing for aneuploidy, assisted hatching, and sperm sorting) and complementary and alternative medicine (such as acupuncture).^5^ Add‐ons are now widely and routinely used in ART cycles and are usually provided at an additional cost.^6^ They are heavily marketed to patients and are used as a key means to distinguish a particular clinic from its competitors.^7^

Despite the widespread use of add‐ons, evidence to support the efficacy and utility of many of them is contested or lacking. Indeed, most add‐ons are not yet supported by high‐quality evidence, and debate about their use is ongoing.^8^ They are also subject to little specific regulation.^9^ Debates about add‐ons have focused primarily on whether simple appeal to patient wishes or desires can justify their use (and, relatedly, what weight antipaternalist considerations should carry)^10^ and whether disclosure, through “traffic light” warnings, for instance, of the limits to evidence of an add‐on's efficacy is sufficient to adequately support ART patients’ decision‐making.^11^ While answering these questions is important, this is not our aim. Instead, we start from a position that focusing merely on the interests, rights, and knowledge of individual ART patients overlooks problematic assumptions to do with information and how it facilitates autonomy.^12^ That focus also fails to acknowledge concerns about the unfettered marketing and offering of ART add‐ons, such as the claim that these practices may be seen as exploitative. To address this gap, we consider the problem of commercial offers of ART add‐ons by using the concepts of hope and exploitation.

Hope for a baby is fundamental to the design and delivery of ART services and is often used as a justification for service provision by ART providers (organizations or physicians offering ART).^13^ Such hope is, for many people, felt to be important to their identity. ART service providers also share their patients’ hopes for success. Consider here a patient, whom we call Mim and to whom we will return later in this article. Having a baby is something Mim has envisaged doing since her own childhood. To achieve this, Mim and her partner have engaged an ART service provider, with whom they have had multiple counseling sessions and built a strong relationship. Mim herself is a health professional, committed to evidence‐based medicine. She is aware that her hopes for the success of ART are reinforced by societal pronatalism, strong preferences for genetic kinship with offspring, and the psychological impact (and possible stigma) of being infertile or remaining childless.^14^ She and her partner have discussed alternatives, such as adoption or remaining childless, and are firm in their decision to proceed with ART, including the use of add‐ons. Mim's hope that she will be able to have a baby through ART is strong yet considered and under her control. Nevertheless, with hope come vulnerabilities, particularly those to do with autonomous decision‐making, the forming of false hopes, and the potential for hope to be exploited.^15^

The third of these vulnerabilities—the exploitation of hope in ART—has gone largely unrecognized. Attending to this necessitates a substantial reassessment of the use of add‐ons because, as we will argue, those who seek to use ART are especially susceptible to the exploitation of their hope for a baby. Crucially, such exploitation can occur regardless of whether the individual is making a fully informed, considered, and autonomous decision to use the offer of add‐ons. This concern therefore undercuts much of the existing debate regarding add‐ons, which has focused primarily on individual autonomous choice.^16^

Our argument is structured into four sections. We begin by demonstrating how ART is a “hope technology”^17^ and then link this phenomenon to existing philosophical conceptions of hope. While our purpose is not to mitigate existing limitations of such conceptions, we aim to show that there are sufficient resources within these accounts to underpin an account of the exploitation of hope for ART patients. We also establish how ART patients, especially those depending on add‐ons, are in a special position of vulnerability and liable to exploitation of their weighty hopes. Next, we argue that, while typical understandings of hope show how it can impair autonomous decision‐making, concerns beyond autonomy—arising from the special position of vulnerability of ART patients—are important too.

We then draw on Jeremy Snyder's recent work to consider exploitation of weighty hopes in the offer of ART add‐ons.^18^ We argue that there is a special relationship between ART providers and patients, in which the providers (grounded in a duty of beneficence) should respect those seeking ART to safeguard their welfare. Marketing and selling unproven add‐ons is a case of exploitation of weighty hopes. In the final section, we address how the risk of exploitation can be mitigated. We do so by examining the adverse nature of exploitation and how it can be prevented—namely, via what Victoria McGeer calls “good scaffolding.” ART providers need to support patients to achieve well‐scaffolded hopes. Although add‐ons may still be offered, this should take place only in a context where the possibility of exploiting hope is actively mitigated against, as with cost‐free offers under a research protocol.

## ART, Hope, and Vulnerability

Hope is a key cognitive and emotional driver of people's attitudes and behaviors when undergoing ART.^19^ Empirical evidence shows that hope influences decisions to commence ART and to continue treatment (especially when treatment failure is unable to be explained), both of which also aim to preclude future decisional regret.^20^ Indeed, ART is labeled a “technology of hope” by Christopher Mayes and colleagues.^21^ In an ethnographic study conducted in the early 1990s of practices of in vitro fertilization in England, Sarah Franklin asserts that the public framing of ART as hope technologies plays an important legitimating role and that hope is inherent to ART and to reproduction more generally. “In the conjuncture between reproduction and technology,” Franklin argues, “are combined two of the most powerful Euro‐American symbols of future possibility: children and scientific progress. It is for this reason that the IVF story is inevitably concerned with the meaning of progress, the character of hope, the desire for children and the will to overcome adversity.”^22^

The phenomenon of hope is particularly important because the probability that a single treatment cycle will lead to a live birth remains low.^23^ Franklin observes, “IVF is described as a ‘hope technology’ because it is the hope it promises, as much if not even more than a ‘successful’ outcome, which leads it to be seen as a desirable option, even when it is expected to fail.”^24^

For ART patients, hope is thus double‐edged.^25^ On the one hand, participants in Franklin's study maintained the hope to have a child. This “hope that keeps you going” was crucial to their continuing ART, even with low success rates.^26^ On the other hand, their hopes also had a “dis‐abling” effect, rendering patients unable to reach the point at which they would cease ART. Indeed, ART patients have reported that they would continue treatment for as long as a chance of success persisted, until they had exhausted all options or their doctor advised them to stop.^27^ This second effect of hope is especially important when it comes to the vulnerability of ART patients (particularly for add‐on use), which we turn to momentarily.

Hope continues to be prevalent in contemporary ART practices. Whether hope is considered using psychological, sociological, clinical, or philosophical approaches, providers of ART clearly have a significant role in fostering it. This manifests in aspects of care such as communication during consultations, as well as in the tone and content of supporting materials like information leaflets and websites, which tend to focus on facilitating people's realization of their desire to have children. Hope for a baby is both an inherent and authentic aspect of patients’ experiences of ART but also a phenomenon that reflects certain cultural attributes, such as pronatalism, and technological imperatives in health.^28^

Although there are numerous philosophical accounts of hope, it has been widely treated as a state of mind oriented toward some desired state of affairs that is believed to be attainable. Contemporary analyses of hope have generated the “standard account,” whereby hope comprises a belief‐desire pair: a person hopes for a state of affairs if and only if she desires it and believes that it has some possibility of coming about, though whether it will is not certain.^29^ In the case of ART with add‐ons, a patient would hope for a live birth in that they desire this and believe that ART offers them the best or only means of manifesting their desire. Hope is, as a result, central to ART; the technology offers the possibility of achieving a desired outcome that would be extremely unlikely to happen in any other way, but that is nevertheless still not guaranteed.

The nature and role of hope also reflect something fundamentally important about why patients place such emphasis on maintaining a hopeful state when pursuing ART and, ultimately, why it leaves them open to exploitation in this area. There are some areas of life that are particularly important or weighty in that they have a significant role to play in one's welfare, sense of self, or core values. The decision to found or expand a family (by whatever means) could readily be taken to be such an area of life. Crucially, the weightier the hope, the more likely it is that individuals will make greater sacrifices, accept greater risks, and make what Snyder calls “leaps of hope”^30^ to realize a desired outcome. Those hoping to have a child are part of a group with especially weighty hopes, and their willingness to make leaps of hope by accepting greater risk and sacrifice increases when those hopes are not met by simpler means. The desire to realize a very weighty hope leads people to pin their hopes on ever‐diminishing possibilities of realization to achieve this outcome, causing those hoping to have a child using ART to take leaps of hope by accepting offers of unproven add‐ons. As this happens, the potential for exploitation of hope increases.

Hope is often treated as a basic human good or positive state in which to exist, on the basis that it offers people a range of psychological benefits when encountering circumstances that seem to frustrate their desires. In the case of ART, the type and nature of patients’ hopes are especially important to them in terms of realizing their significant life choices.^31^ As mentioned above, the weightiness of the hope to have a child increases patients’ willingness to take risks with unproven procedures but also disposes them against questioning the rationale behind the offer of those procedures. This places patients in a special position of vulnerability (vis‐à‐vis ART providers) that is sufficient to raise considerable ethical concern. Moreover, as we go on to argue, this concern persists even when ART patients know they are essentially buying hope (or, at least, buying an extension to their existing hopes).^32^

In making such a claim, we first need to clarify our use of “vulnerability.” Previous work has established the importance of treating vulnerability not as an inherent property of individuals per se but rather as qualified, so that a person can be understood to be vulnerable only *in terms of something*.^33^ According to this view, while there may be some aspects of vulnerability that can be deemed to be positive aspects of the human condition (such as a sense of shared experience with others in the same situation), our concern is that ART patients like Mim have a particular vulnerability because they may experience a range of harms: physical, psychological, emotional, financial, and social. We also claim that ART patients are vulnerable in a more fine‐grained sense: *sustaining harm arising from their hopes for a baby* and from their relationship with their ART provider, even if they are empowered and aware of their hopes. Indeed, the vulnerability of ART patients is an inherent aspect of their relationship with their provider (even if this is good): they are entrusting them to fulfill their hopes for a baby. Such vulnerability, in turn, generates obligations on the part of the provider to safeguard the welfare of the patient. We focus our subsequent discussion on what may be termed “the exploitation of vulnerability.”

Several additional factors come into play when considering the vulnerability of hopeful patients seeking ART. One is the concern that the hope to have a baby can sometimes be so profound as to overwhelm or override both decision‐making processes (such as a decision to cease ART or to decline an offer of an add‐on) and other factors (such as personal welfare). As we discuss in the following section, insofar as ART patients are dependent upon the success of such technologies to realize their hopes of having children, they are especially vulnerable to what Adrienne Martin has identified as “autonomy‐impairing hopes”^34^ and McGeer has termed “wishful hoping.”^35^ However, even where these threats to autonomy are overcome, there remains an additional vulnerability to exploitation of the ART patient's hopeful state. ART patients are therefore in a special position of vulnerability vis‐à‐vis the reproductive technologies they rely on to realize their hopes to have children.

In this regard, it is also important to consider another aspect of hope that is commonly raised as a point of concern, namely, *false hope*. According to the standard account of hope, where belief in a desired outcome is seen as probable, false hope occurs where an individual bases their hope on a misunderstanding of the likelihood or possibility that the hoped‐for outcome will arise. False hopes, like other forms of hope, can be exploited. Intentionally creating false hope in another individual is widely seen as a serious moral wrongdoing because it involves a deliberate intention to deceive, either through actively misleading or through deliberate omission of relevant information. However, false hope can also arise in other ways, such as through engaging in a form of willful blindness to probable outcomes or even simply refusing to engage with such information. We return later to the role that false hope might play with ART patients and the commercial offer of add‐ons.

## Commercial Offers of Add‐Ons and Autonomy‐Impairing Hopes

Although many of the available accounts of hope are grounded in the same “standard” account we outlined above (that is, hope is primarily a combination of belief and desire), they can be further refined. A key kind of hope relevant to our purposes—“autonomy‐impairing hope”—has been discussed by Martin in the context of terminally ill patients who are offered experimental yet potentially life‐saving treatments.^36^ She recognizes the importance and role of hope in such situations, particularly in the face of very low probability of success. This kind of hope is, we contend, also suited to understanding concerns over hope in ART.^37^ While ART differs from terminal illness because, among other reasons, it involves a life‐giving rather than a life‐prolonging decision, it nevertheless has significant psychological and financial implications, as well as high morbidity for some patients (for example, due to ovarian hyperstimulation).

Martin begins by offering a minimalist conception of hope:

- “Hope for an outcome involves the *desire* for [that outcome].”
- “Hope involves *imaginative engagement* with the desired outcome, such as prayer, mental imaging, or fantasizing.”
- “Emotions, including hope, play a *framing role* in relation to our uptake, interpretation, and deliberative use of information.”^38^

Martin argues that the role played by the second and third above points, namely, hopeful imaginative engagement with a desired outcome and the emotional framing therein, can enhance autonomy. However, these elements of hope can also impair autonomy, especially when such emotions are strong. She states, “Since emotions can affect information processing, they can make a person's decisions more or less autonomous. That is, an emotion—especially a strong emotion, such as the hope for unlikely cure—can shape a person's deliberation in a way that is more or less consistent with her own ideas regarding what counts as good deliberation.”^39^

Recall the case of Mim. After three unsuccessful full cycles of IVF, Mim discusses with her clinician whether there might be any further options to try. One such option is to include two add‐ons in subsequent cycles: an endometrial scratch and embryo glue. Mim receives, and understands, information that there is not yet evidence that these add‐ons increase the chances of a live birth. She also understands that these add‐ons have the potential to cause harm to her. However, Mim also really wants to become a parent and is “willing to try anything” to achieve this goal. She strongly hopes that including these add‐ons in her next cycle will increase her chance of having a baby.

On Martin's account, Mim's hopes for a baby could be autonomy enhancing or autonomy impairing. On the one hand, her hopes could be said to enhance her autonomy if they motivate her to continue to use a recommended ART add‐on when she might have otherwise stopped using it. While she is aware of the equivocal evidence, she is confident that she has deliberated well and decides based on her considered reflections. She resolves that it is still worth using the add‐on despite its low chance of success (or even potential for harm) because those downsides are more than off‐set by her strong hopes for a baby, so that achieving this hoped‐for outcome justifies taking the potential risks. On the other hand, Mim's hopes could impair her autonomy if they lead her to downplay the status of the evidence (say, if she convinces herself that the add‐on is better than it is), to dismiss the possibility that the add‐on will cause harm, or to make a decision without sufficient deliberation. Hence, were Mim to seize the offer of the add‐on heedless of the low likelihood of its success because it corresponded with her hopes for a baby, and realizing these hopes were all that mattered to her, that would be an indication that her hopes were impairing her autonomy. Although the difference between autonomy‐enhancing and autonomy‐impairing hopes can be subtle, it is important to recognize that hope can impair an ART patient's ability to make autonomous decisions.

Martin concludes (albeit in the context of her terminal illness example) that the possibility of autonomy‐impairing hopes is high enough in offers of experimental treatments to warrant taking explicit steps to avoid exploiting those who hope for unlikely cures. Concerningly, ART patients report that they value evidence about add‐ons yet still use them.^40^ It is therefore important to pay attention to the possibility of autonomy‐impairing hopes in the offer of ART add‐ons.

That said, while Martin's account captures some important features of hope and helps explain the psychological state of an individual hoping for a particular outcome, it effectively reduces concerns about hope and exploitation to concerns about autonomy. It thus offers a narrow analysis of the vulnerability of ART patients being offered add‐ons, as it overlooks another important risk associated with hope: that one could be entirely well informed and autonomous yet still have one's hope exploited. To capture this, in the following section, we construct an account of what it is to exploit ART patients’ hopes.

## Hope and Exploitation in the Offer of Add‐Ons

Snyder's recent in‐depth account of what it is to exploit hope^41^ is useful in considering add‐ons in ART. Snyder follows Martin in recognizing that the levels of thinking, fantasizing, planning, and engaging emotionally with a desired outcome are all crucial elements of hope, particularly for highly valued (“weighty”) outcomes. However, accounting for what hope *is* tells one only half of what is necessary to understanding the exploitation of hope. One also needs to capture *how* hope can be exploited. To achieve this, Snyder proposes a novel account of the exploitation of hope, which proceeds on the basis that health care providers have a specified duty of beneficence toward patients. This duty arises because patients entrust their welfare to their health care providers. It goes beyond what consent or any general duty of beneficence establishes, incorporating the vulnerabilities patients have due to their substantial and weighty hopes for success in a treatment. The situation of entrustment in these circumstances is referred to by Snyder as “moral entanglement.”^42^ It forms a particular type of relationship and can create both special obligations and power imbalances.^43^

To begin to understand what is so special about these moral entanglements and resulting specified duties of beneficence, it is important to recognize that individuals who hold weighty hopes are liable to take risks—what Snyder terms “leaps of hope”—to try to fulfill them. Part of taking these risks involves the process of partially entrusting their welfare to third parties who offer means to achieve their weighty hopes. The entrustment is partial because it involves entrusting only certain specific aspects of an individual's well‐being to another.^44^ The trustee is given discretion over how to care for something that is particularly valued by this individual, which generates a specified duty of beneficence to care for that individual arising within the scope of their interaction or relationship. The liability to take risks is enveloped in the third parties’ specified duty of beneficence toward the individual as an obligation to safeguard their welfare against vulnerabilities arising from hopeful risk taking. Such a duty is apparent with respect to ART whenever patients entrust providers to source and perform treatments. Exploitation can then arise when, in the context of this important relationship, a specified duty of beneficence is not fulfilled. Instead, providers may use the relationship of entrustment as an opportunity for gain without fulfilling the responsibilities for safeguarding patient welfare.

Crucially, this account of exploitation can hold even when a patient is fully aware that the proposed treatment is unlikely to confer any real benefit or when it costs more than they might be able to reasonably afford.^45^ This means that exploitation cannot be reduced to a failure by the ART provider to supply proper information and evidence for the nature and efficacy of the add‐on, nor to a failure of autonomy by the ART patient. Therefore, we contend below that even accounts of exploitation such as Alan Wertheimer's^46^ (which hold that it can occur even in the context of mutual consent) are not sufficient to capture the moral wrongness of the exploitation. Instead, the exploitation occurs because the provider of the medical intervention treats the patient without sufficient respect, the need for which arises from the duties corresponding to the special relationship between the provider and patient. So, such exploitation arises not only in cases when an ART patient autonomously decides to pursue add‐ons and is aware of their limitations; it can also arise where there is no obvious harm. This includes cases in which an ART patient has more than enough resources to purchase the unproven add‐on or in which they can justify the cost of the intervention because it helps them to actively maintain a hopeful attitude by “doing something rather than nothing.” The exploitation occurs because ART providers, in selling add‐ons, are benefiting from the weighty nature of the hopes of the patient to pursue the possibility of a baby in a way that is unlikely to result in any real benefit.

Some may argue that no exploitation takes place when an ART patient is simply making a rational choice—regardless of evidence—to purchase hope for a better future life by using their resources (time, money, and so on) in a way they see fit. To fully understand why this argument does not hold, we need to look more closely at Snyder's account of the exploitation of hope. Snyder understands exploitation as a failure to respect. On this view, failure of respect (a respect for persons, grounded in a specified duty of beneficence) arises from the nature of the relationship between the ART provider and the patient. In such relationships, a duty of beneficence arises because of partial entrustment—because the patient entrusts the ART provider with specific aspects of their health and well‐being. Further, the forming of such relationships generates a form of moral entanglement or emotional vulnerability, whereby these special obligations to safeguard an individual's welfare are generated. Such entanglements are particularly common in medical contexts, where patients regularly entrust all sorts of aspects of their well‐being to providers, such as bodily autonomy, in the expectation of greater overall care for their well‐being. In the case of ART, it is not simply that a patient is entrusting their reproductive health to the ART provider. The known nature of ART, the moral entanglement, the disposition to take risks through leaps of hope, are all relevant aspects of patient well‐being that fall within the scope of the partial entrustment. Other medical contexts might also give rise to far more general cases of entrustment. For example, a patient seeking help with an undiagnosed condition who presents to their primary physician might consider all aspects of their health and well‐being to fall within that physician's remit. Hence, what is at stake is not a simple prima facie duty of beneficence, something that might be met by overriding competing duties. The moral entanglement *creates* a duty of beneficence that goes beyond the standard duties of a medical relationship. Here, duties are owed to persons, and a provider's failure to act in a patient's best interests regarding relevant aspects of that patient's welfare entrusted to them is a failure of respect owed to that person.

The patient‐provider relationship in offers of add‐ons is an important example of how such a partial entrustment arises. This relationship is key to understanding the wrongness of exploiting hope in the context of ART. If the patient is aware that the intervention is unproven yet requests it anyway, they must be keen to take control of their well‐being. As those offering unproven add‐ons to ART cannot offer a substantiated likelihood of a live birth, they are promoting a wider sense of well‐being that includes nurturing the benefits of hope. Therefore, ART patients are entrusting not only their fertility care to ART providers but also the aspects of their well‐being that are linked to their hopeful state. This creates a substantial moral entanglement between provider and patient, with ART providers knowingly offering an intervention that is based on promoting this wider sense of well‐being. The obligations arising from the patient‐provider relationship mean that safeguarding patients’ well‐being (in relation to their hopeful state) becomes a specified element. To disregard the obligations that arise in relationships where one is fostering hope would therefore be a case of exploiting these individuals’ hopes. This is because, if an ART provider fails to meet their special obligations to safeguard well‐being in a relationship of partial entrustment (upon which the offer of ART add‐ons is based), they are no longer respecting the patient as an individual. Instead, they are treating the patient as a mere means for likely financial gain.

Under Snyder's account, the same responsibilities not to exploit hope hold whether a hope is genuine or false. It may well be that, because the likelihood of having a baby would (by definition) be smaller in the case of false hope, the individual's vulnerabilities are higher. Thus, there is a more demanding obligation (within a relationship of partial entrustment) to ensure that one should safeguard an individual from forming such false hope. While it is possible to have a mutually beneficial relationship of supporting a hopeful patient and benefiting from their hope in the intervention, being sufficiently transparent to maintain realistic hopes in light of limited evidence of efficacy is extremely difficult. Indeed, such relationships are likely to lead to exploitation, especially in cases where there is a desire (or need) to market and profit from these interventions.

## Understanding and Mitigating the Exploitation of Hope in ART

We have considered several ways in which ART patients’ hopes can give rise to vulnerabilities when patients are offered add‐ons. This can occur in cases ranging from ones in which hopes are (in certain ways) autonomy impairing to ones in which the nature of the patient‐provider relationship opens hopes to the risk of exploitation. Two main kinds of awareness are needed in considering what might be done to mitigate these concerns: one is a recognition of the adverse impact the exploitation of hopes has on patients deliberating about undergoing ART, and the second is an understanding of how refraining from marketing and selling add‐ons can appropriately scaffold ART patients’ hopes.^47^ In identifying the need for this recognition and understanding, we do not intend to imply that innovation is inappropriate in the context of ART—on the contrary, it is important. But the development and offer of innovative interventions in ART need to be undertaken while providers also take all possible steps to avoid exploiting their patients, who are in a special position of vulnerability.

### Recognizing the adverse impact of exploiting hopes surrounding patient deliberations.

Above, we introduced how Martin considers the effects of strong hopes on an agent's deliberative capacities to be potentially autonomy impairing, thereby creating a particular type of vulnerability among ART patients like Mim. This leaves them open to potential exploitation because the nature of these hopes downplays the status of evidence about the efficacy of add‐ons. Martin also draws attention to what we call the “generative nature” of such hopes. Hope can be generative in that it grows and develops, encouraging people to pursue the object of their hope. This pursuit in turn leads to a further bolstering of that hope and the development of means to achieve it.

Generative hopes can have a range of effects on the hoper.^48^ Possession of such hopes can support the development of traits such as tenacity, focus, determination, and so on as a means to achieve the hoped‐for goal. However, it can also lead to an excess of longing, overconfidence, stubbornness, or even a decline into a wishful passivity. For example, people with strong emotional investments in unlikely outcomes are “all too ready to seize upon any proffered hope”^49^ that accords with what they desire. This generative disposition toward hopefulness, Martin argues, is exacerbated when representatives of institutions are offering such hopes. She observes, “[T]he word ‘hope’ is *almost magical*: a researcher who says it to a potential participant could hardly choose a better way to encourage her to ignore the risks and burdens of participation, play up the benefits, or even imagine benefits the researcher has no right to offer.”^50^

We contend that the selling of unproven ART add‐ons can lead to generative hopes with effects that leave the individual with them vulnerable, making them liable to exploitation. Two kinds of generative hopes arising in this area are false hopes and hopes that are maintained despite a clear grasp of an evidence base indicating the low likelihood that the desired outcome will manifest. As we indicated earlier, false hopes can arise where the likely efficacy of the preferred treatment is misunderstood or misrepresented. While misrepresentation or perpetuation of a misunderstanding is straightforward deceit, generative false hope can still arise where ART patients are provided with accurate information about add‐ons. The strong emotional investment in the success of the intervention can generate a false belief that the probabilities of success are greater than they actually are.

The second form of generative hope arises when the patient is entirely clear about the likely efficacy of the add‐on but hopes so strongly to have children that they are willing to try anything. Their hope, therefore, is not based on a false belief about the probability of success but rather on prioritizing the desire to continue to attempt to do something positive toward achieving the hoped‐for outcome. In essence, the patient wishes to maintain hope and therefore sees even a low chance of success as contributing to maintenance of a hopeful state.

Exploitation of generative hopes can occur in two ways. First, it can happen when a patient forms and acts on a hope to their significant detriment in terms of financial costs or other harms, such as a negative effect on their physical or psychological health or family life. This would result from an ART provider's failure of respect (on Snyder's account); by allowing clear harms to manifest—harms not offset by a justifiable likelihood of the desired outcome—the provider would not be fulfilling their specified duty of beneficence. Second, exploitation can occur even where there is no obvious harm. An ART patient may have more than enough resources to purchase the unproven treatment, or they may see the positive aspects of actively maintaining a hopeful attitude—often described as “doing something rather than nothing”—as genuinely worth the cost it gives rise to.

This second sense of exploitation generates an interesting concern about the exploitation of hope. As mentioned, one could claim that it is rational for an ART patient to purchase hope for a better life using their resources as they see fit.^51^ This would cohere with Martin's views about there being autonomy‐enhancing hopes that motivate people to act in positive ways. Accordingly, one may argue that this does not exploit generative hopes but simply allows autonomous agents the freedom to act and to gain the benefit they find in pursuing their hope for a desired outcome.

A strong focus on respecting autonomy in ART provision has meant that considerations about exploiting hope have stopped at this point. However, it is still possible to account for there being exploitation of hope even in these cases. The answer once again lies in terms of viewing exploitation as a failure to respect, which can occur even in the absence of an overtly harmful action.

The commercial provision of ART may further entrench this problem. Where ART add‐ons are being marketed and where profits or returns to investors are desired, exploitation of hope is more likely. The mere fact of being expected to turn a profit can affect even the best‐intentioned ART provider's efforts to support realistic hope in a nonexploitative way.

To understand why exploitation can occur even when an add‐on is rationally chosen, it is useful to draw on McGeer's concept of “scaffolding.”^52^ McGeer regards hope as being dependent upon the quality of what she terms the “scaffolding” of one's hopes by others, particularly if such hope is regarded as generative in the sense we have outlined. This scaffolding involves a process of staged support of meaningful development, which, in turn, gives rise to enhanced agency. The goal is to facilitate people's ability to self‐scaffold, but such processes can take time and need continued support. McGeer goes on to argue that the act of hoping, particularly in cases of weighty hoping, requires other people to be responsive to the hopes, as this scaffolds the continuation of that hope. This is exactly what is needed in cases of generative hope, as “good scaffolding” encourages a reliance on one's own abilities through what McGeer describes as “responsive hope.”

Responsive hope is felt by people who imagine themselves as capable of bringing about a future that they want to inhabit, even if such a future is not what they had originally hoped for. They achieve this by developing a capacity to engage with real‐world constraints on the possibility that a desired outcome will transpire; hence, they are “responsive” to the world as it is. Responsive hope is supported by “peer scaffolding,” through which the individual who has an established ability to self‐scaffold is stimulated and encouraged in their capacity to realize their hopes by a community, by other people. Such good scaffolding, enabling this responsive hope, is thereby autonomy‐enhancing because it offers a supportive and respectful mechanism for an individual to develop a self‐directed agency, rather than becoming dependent upon others to bring about an outcome.

Conversely, poor scaffolding encourages an underreliance on one's own abilities, through what McGeer terms “wishful hoping.” This is a concern when engaged in any form of generative hoping, as it encourages a more passive attitude and dependence upon the external world for the fulfillment of one's hopes, rather than a realistic understanding of whether or how they might be realized. As a result, an agent is unable (or unwilling) to imagine another future if their hopes are frustrated. Such “poor scaffolding,” leading to wishful hope, is thereby autonomy impairing, as there is very limited development of one's own agency and a strong overreliance on the external world to satisfy one's desires and hoped‐for outcomes.

Following McGeer, we might say that ART providers not only provide hope to their patients through the technologies they offer but that they serve to scaffold that hope as well. ART patients are susceptible to poor scaffolding, leading to wishful hoping, due to their reliance upon reproductive technologies to have a baby. (The hope Franklin analyzed as “dis‐abling” might be seen as a form of wishful hope.) The hope imperative in ART encourages dependence on this intervention—and, in contemporary ART, the use of add‐ons—to fulfill the hope for a baby. The relationship between ART providers and patients thus generates a responsibility for providers to mitigate against autonomy‐impairing wishful hoping and to support well‐scaffolded, autonomy‐enhancing approaches to generating hopeful states among their patients.

### Mitigating the possibility of exploitation through regulating add‐on use.

How, therefore, might ART patients’ hopes be appropriately scaffolded so that they might continue to grow and develop in a nondetrimental manner for the hopers and leave these patients less open to the possibility that their hopes will be exploited? To provide good scaffolding, ART providers must not market or sell add‐ons. This position derives from obligations arising from the relationship between patients and providers. Because the exploitation of ART patients’ hopes could take place even in the context of the best clinical intentions and clear communication as to the limits of add‐ons, ART providers should proactively mitigate this vulnerability by refraining from selling add‐ons.

The requirement to refrain from selling add‐ons raises the question, posed by Wertheimer,^53^ as to whether consensual and mutually advantageous transactions should be prohibited by society, even when exploitation is present. Although fully responding to such concerns is beyond our scope here, a brief response is warranted. Wertheimer's transactional account of exploitation has a very different moral basis than the respect‐based account we are using. A primary motivation for employing an account such as Snyder's is the recognition given to a specific duty or obligation having been entered into. This makes it particularly apt for our understanding of the wrongness of the exploitation in the selling of unproven add‐ons because of the very specific obligation (arising from moral entanglement and grounded in the specified duty of beneficence) owed by the provider toward the patient. A violation of this obligation constitutes an important moral wrong. This wrong is absolute, and not ameliorated by expediency, as in the case of transactional mutually beneficial yet exploitative accounts.

Furthermore, this is also not to say that ART providers must completely refrain from offering all add‐ons. Rather, the contexts in which it is ethically permissible to *offer* them should be restricted to those for which the risk of exploiting hope is actively mitigated. One of the clearest ways to achieve this would involve both not charging for add‐ons and providing them in such a way as to contribute to the evidence base (under a suitable protocol, with relevant approvals in place) about their efficacy and utility. Should evidence of their efficacy be established, concerns about exploitation would radically diminish, and a return to their commercial provision may be warranted.

Debate continues regarding what type of evidence will resolve debates over the efficacy and utility of add‐ons,^54^ and it is beyond the scope of this paper to detail what should constitute appropriate evidence or how this should be obtained. However, we note several options in the literature: a randomized controlled trial, prospective cohort studies, individual patient‐data analysis,^55^ and context‐based care.^56^ We also recognize that there will be impediments to these approaches, such as the cumbersome and time‐consuming nature of research ethics and governance approvals, financial costs, and the fact that many commercial providers may not be active in research as a matter of course. However, these issues are not unique to ART; rather, they provide further impetus for research and innovation ethics processes to be reformed and for professional societies to better support their accredited members to practice health care while contributing to the evidence base and then to draw on high‐quality evidence in the care they provide. Offering add‐ons under a suitable protocol designed to improve the evidence base of their effectiveness will mitigate exploitation of hope and will uphold providers’ duty of beneficence.

It may be objected that, given everything we have discussed about the nature of weighty hopes and challenging aspects of their generative nature in the context of ART treatment, ART providers might still be exploiting patients by recruiting them into trials or studies of the benefits of add‐ons. Such a concern has two elements. One is that the weighty nature of ART patients’ hopes is such that they will simply agree to be recruited into such activities, particularly if they would have previously paid to access such add‐ons with the same evidence base as to their efficacy. The second is that, in recruiting patients for research, ART providers will ultimately still be exploiting the hopeful states of these patients because they will be benefiting from the study findings in terms of identifying those add‐ons that have a greater degree of efficacy that they can then market and sell.

McGeer's account of hope and scaffolding is helpful again here. Good scaffolding engages and generates hopers in a form of responsive hope, which allows them to actively participate in bringing about a future they want to inhabit. We argue that ART providers, by encouraging a dependence on their primary treatments and add‐on interventions, generate a poor scaffolding that leads to wishful hoping. The possibility of participating in an endeavor like an appropriately constructed study, however, has the potential to offer an entirely different form of scaffolding for ART patients’ hopes. Participation in a well‐run study would be more likely to engage ART patients in the very mechanism needed for bringing about the success of their hoped‐for outcome. By promoting active engagement, rather than simply being passive recipients, providers could offer a good scaffolding for patients’ hopes to be generated, leading to a responsive type of hope. So, while patients might be entering such activities with a significant set of weighty hopes for a successful outcome, these hopes would now be well scaffolded, and the ART providers running the trials would thus be supporting rather than exploiting patients’ hopes. ART providers might also be fulfilling a further, albeit more general, duty of beneficence in that they have a responsibility to obtain better evidence about the use of add‐ons and to make that transparently available.

## Ethically Scaffolding Hope in ART

People who hope to have children via the process of gestation and birth and who are not able to realize this via sexual intercourse do not pursue ART frivolously. Rather, they seek to use these reproductive technologies because they are (or perceive themselves to be) dependent on them to fulfill their hopes of parenthood. This is a vulnerable place to be, as the nature of these hopes are weighty both in terms of their life significance and because they lead individuals to make “leaps of hope” that leave them liable to significant exploitation.

We have argued that avoiding the exploitation of hope necessitates that ART providers abstain from selling any nonessential and unproven treatments to their patients. This is a provision against *selling* such treatments, on the grounds that the demands of safeguarding against exploitation of hope would be virtually impossible to meet where a consumer relationship exists between patient and health care provider for unproven treatments. There remains, however, the possibility of offering add‐ons under strict conditions, for example, a research‐based intervention with the intention to produce high‐quality evidence to inform future practice. In such a setting, hopes and welfare would still need to be managed so that exploitation was not an issue.

Assisted reproductive technologies are hope technologies. They not only give hope to the individuals who use them, but they also sustain the collective hope that advances in medical science will allow humans to overcome adversity. The promotion of hope is tied to innovation, and there are both commercial and consumer pressures to be competitive in the field of for‐profit ART. Untrammeled innovation, however, can generate harm. ART providers must abstain from exploiting hope by not selling untested add‐ons. Doing so will allow them to meet the obligations that arise from being in a trusted position as a provider of fertility care.

## Acknowledgments

This work was funded through the National Health and Medical Research Council of Australia, through award APP1181401.

The authors thank Miriam Wiersma and the conflict of interest in ART research team for their invaluable input during the writing of this paper.

## Disclaimer

The views expressed are those of the authors and not of all project investigators.
